# Chemical diversity and species differentiation in Brazilian *Vanilla*: insights from LC-HRMS/MS metabolomics

**DOI:** 10.1007/s11306-026-02422-8

**Published:** 2026-03-28

**Authors:** Gesiane S. Lima, Giovanni B. Bevilaqua, Hugo G. Machado, Rosa B. N. Alves, Luciano B. Bianchetti, Lanaia I. L. Maciel, Bianca M. M. G. Acioli, Nerilson M. Lima, Roberto F. Vieira, Boniek Gontijo, Gabriel F. dos Santos

**Affiliations:** 1 Institute of Chemistry, Federal University of Goiás, Goiânia, GO 59078-970 Brazil; 2 Department of Biological Sciences, State University of Santa Cruz, Ilhéus, BA 45662-900 Brazil; 3 Embrapa Recursos Genéticos e Biotecnologia, Parque Estação Biológica, Distrito Federal, Brasília, 70770-901 Brazil; 4 Institute of Chemistry, Federal University of Alfenas, Alfenas, MG 37130- 001 Brazil

**Keywords:** *Vanilla*, Metabolsomics, LC-HRMS/MS, *Vanilla pompona* Schiede, *Vanilla phaeantha* Rchb.f., *Vanilla calyculata* Schltr.

## Abstract

**Introduction:**

*Vanilla* species represent a taxonomically complex and economically important group of orchids, yet species discrimination and chemotaxonomic characterization remain challenging due to high intrageneric metabolic variability and limited molecular insights.

**Objectives:**

We aimed to apply an untargeted metabolomic approach to characterize the metabolic signatures of three Brazilian *Vanilla* species (*V. pompona*,* V. phaeantha*, and *V. calyculata*) and to evaluate the relative contributions of species identity and biome origin to metabolic diversification.

**Methods:**

The leaf metabolome of 102 *Vanilla* were profiled using high-resolution LC-HRMS/MS. Multivariate analyses including PCA and PLS-DA served to explore variance structures and build discriminant models. Discriminant features were selected using integrated criteria (VIP scores and volcano-plot significance). Annotation leveraged GNPS molecular networking and in silico databases to classify key metabolite classes.

**Results:**

Unsupervised PCA revealed pronounced intraspecific metabolic heterogeneity, preventing spontaneous grouping by species or biome. Supervised PLS-DA models, however, provided robust species classification (Q² = 0.74–0.90), while biome-based models lacked predictive power, indicating limited environmental influence on leaf metabolomes. Consensus selection between models identified 17 core biomarkers, including phenolic acids, cinnamic acid derivatives, *C*-glycosylated flavonoids, lipids, terpenoids, and N-containing compounds, with ferulic acid (a key precursor of vanillin) emerging as a prominent discriminating metabolite.

**Conclusions:**

Untargeted LC-HRMS metabolomics coupled with chemometric modelling delineates species-specific metabolic fingerprints within *Vanilla*, offering a generalizable strategy for chemotaxonomy and species authentication. The conserved metabolic features also spotlight biologically meaningful pathways for biodiversity assessment and valorization of native *Vanilla* resources.

**Supplementary Information:**

The online version contains supplementary material available at 10.1007/s11306-026-02422-8.

## Introduction

The genus *Vanilla* Mill. (Orchidaceae) comprises over 100 species distributed across the tropical regions of Africa, Asia, and the Americas (Pansarin & Menezes, 2023). In Brazil, this genus is represented by more than 40 species (Pansarin, 2025). Despite this diversity, the delimitation of species within *Vanilla* remains poorly understood, and several Brazilian taxa are currently synonymized. This taxonomic uncertainty may result in a misestimation of species’ geographic distributions (Koch et al., 2013; Pansarin, 2024), limiting our understanding of their true diversity and hindering efforts to conserve and sustainably exploit *Vanilla* genetic resources.

Among the species within this genus, *Vanilla planifolia* is the most extensively cultivated and commercially traded, accounting for the majority of global vanilla extract production. *Vanilla tahitensis* J. W. Moore and *Vanilla pompona* Schiede are known to synthesize distinct aromatic compounds that confer unique flavor profiles to commercial products (de Oliveira et al., 2022). Several other *Vanilla* species also produce aromatic molecules and other secondary metabolites of economic interest (Chen et al., 2025; dos Santos et al., 2024), and the increasing global demand for natural vanilla extract has stimulated the exploration of alternative species as potential sources of valuable compounds. Accurate differentiation among these species is therefore essential for preserving biodiversity, ensuring quality control, and preventing commercial fraud (Chen et al., 2025; Krueger & Krueger, 1985). Beyond these aspects, reliable species identification provides critical insights for conservation strategies, supports the development of sustainable cultivation systems, and promotes the biotechnological exploitation of *Vanilla* species as promising reservoirs of aromatic and bioactive compounds (Flanagan et al., 2022; Hu et al., 2019; Nadal et al., 2025).

A key component of *Vanilla*’s aromatic profile is vanillin, which represents 1–8% of the dry weight of the pods and accounts for approximately 80% of their total aromatic compounds (Perera & Owen, 2010; Zhang et al., 2014). Beyond its well-known role as a flavoring and fragrance agent, vanillin also exhibits antimicrobial and antioxidant properties (Wang et al., 2024), making it a valuable natural preservative in food and cosmetic applications (Brunschwig et al., 2009; Ciriminna et al., 2019; Tomadoni et al., 2016). This reinforces the importance of characterizing the metabolic diversity among *Vanilla* species, since variations in vanillin content and the presence of other bioactive metabolites can greatly influence the sensory, commercial, and functional properties of *Vanilla* products (de Oliveira et al., 2024). Therefore, a comprehensive and accurate taxonomic framework is fundamental to fully explore the economic, ecological, and biotechnological potential of this diverse genus.

In this context, metabolomics has emerged as a powerful approach for the comprehensive chemical characterization of plants, enabling the differentiation of species based on their metabolic profiles (Salem et al., 2020). Liquid chromatography coupled with mass spectrometry (LC–MS) is one of the most widely employed techniques in metabolomics, offering high-resolution separation and accurate identification of both primary and secondary metabolites (Phapale et al., 2020; Xiao et al., 2012; Zhou et al., 2012). This approach is particularly valuable for analyzing the complex chemical composition of plant species, as it enables the detection of bioactive compounds such as alkaloids, flavonoids, and phenolic derivatives (Álvarez-Fernández et al., 2015; Lima et al., 2025; Niculau et al., 2016). Additionally, LC–MS could facilitate the differentiation of taxa by generating comprehensive metabolic fingerprints, which can be further explored using multivariate statistical analyses and structural annotation tools to identify species-specific biomarkers (Kharazian et al., 2025; Worley & Powers, 2012).

In this study, we apply metabolomics-based approaches using LC–MS to explore the metabolic diversity of Brazilian *Vanilla* species, aiming to differentiate taxa through their distinctive chemical signatures. By combining LC–MS information with chemometric data analysis, we seek to identify key metabolic markers that support accurate species classification, quality control, and the authentication of vanilla-derived products. This integrative approach advances the understanding of *Vanilla* phytochemistry and highlights the potential of metabolomics as a powerful tool for biodiversity assessment, conservation planning, and the sustainable exploitation of *Vanilla* genetic resources.

## Materials and methods

### Chemicals and materials

HPLC-grade methanol was obtained from Tedia Company (Fairfield, USA). Formic acid and caffeine-(trimethyl-^13^C_3_) were acquired from Sigma-Aldrich (St. Louis, USA), while progesterone-*d*_9_ was supplied by CDN Isotopes (Quebec, Canada). Ultrapure water, with a resistivity of 18.2 MΩ·cm, was produced using a Master System MS2000 water purification system (Gehaka, São Paulo, Brazil).

### Sample preparation

A total of 102 different *Vanilla* plants were analyzed, comprising fifty from *V. pompona* Schiede, thirty-five from *V. phaeantha* Rchb.f., seventeen from *V. calyculata* Schltr. The plants were collected from different locations in Brazil (representing different biomes: Cerrado, Amazon, Atlantic Forest, and Caatinga) and cultivated in a greenhouse at the Embrapa Genetic Resources and Biotechnology in Brasília, Federal District, Brazil. All information regarding species, biomes, and the number of samples are presented in the supporting information, Table S1.

The Amazon occupies 49% of Brazil, is predominantly located in the Northern Region, and has an equatorial climate with temperatures of 25–28 °C, high humidity, and abundant, well-distributed rainfall; soils are nutrient-poor and intensely leached, with fertility concentrated in the biomass. The Cerrado covers ~ 23.3% of the national territory and is the largest tropical savanna in South America, occurring mainly in central Brazil, with a seasonal tropical climate (dry season from May to September and rainy season from October to April), temperatures of 22–27 °C, and old, acidic, highly weathered, nutrient-poor soils. The Caatinga, exclusively Brazilian and the most biodiverse semi-arid region worldwide, presents a semi-arid climate with temperatures above 25 °C, scarce and irregular rainfall, and prolonged droughts; soils are generally shallow and stony, sometimes relatively fertile, but prone to desertification and erosion. The Atlantic Forest extends across 17 states along the Brazilian coast and has a tropical humid climate, high rainfall and humidity, variable temperatures, and ocean-influenced precipitation; soils are typically deep and weathered, potentially fertile, yet prone to erosion (de Queiroz et al., 2023; IBGE. Instituto Brasileiro de Geografia e Estatística, 2019).

The plant material is registered in the Sistema Nacional de Gestão do Patrimônio Genético (SisGen) under code A301AFD. Fresh leaves from each plant were collected, cleaned, and freeze-dried to complete dryness using a Lyotech Freeze Dry System LS 3000 (São Carlos, Brazil) for 48 h. The dried leaves were then ground into a fine powder using an electric grinder and stored at −20 °C until further analysis.

The extraction procedure followed the method described by Vos et al. (De Vos et al., 2007). Briefly, 50 mg of the ground plant material was extracted with 1 mL of a cold methanol: water solution (75:25, *v/v*) containing 0.1% formic acid. The extraction process involved 10 s of vortexing, followed by 15 min in an ultrasonic bath, and concluded with centrifugation at 15,000 rpm for 10 min. The resulting supernatant was filtered through a 0.22 μm membrane filter and transferred to an HPLC vial (280 µL). Before injection, the samples were spiked with 20 µL of a solution containing caffeine-^13^C_3_ and progesterone-*d*_9_ at an individual concentration of 50 µg mL⁻¹, resulting in a final concentration of 3.57 µg mL⁻¹ for each internal standard. For quality control (QC) samples, 10 µL aliquots from each of the 102 samples were pooled to generate a representative mixture, which was used to monitor instrument performance, ensure data consistency, and facilitate normalization during metabolomic analysis.

### LC-HRMS/MS analysis

LC-HRMS/MS analyses were performed using an HPLC 1220 Infinity II (Agilent Technologies) coupled to a Q-Exactive hybrid Quadrupole-Orbitrap high-resolution mass spectrometer (Thermo Scientific) equipped with an electrospray ionization (ESI) source. Separation was achieved using a Luna Omega Phenomenex C18 column (4.6 × 150 mm × 5 μm; Agilent) with a gradient elution program. The mobile phases consisted of (A) water with 0.1% formic acid and (B) methanol with 0.1% formic acid. The gradient started at 15% B, held for 4 min, increased linearly to 100% B over 15 min, maintained for 15 min, returned to initial conditions in 1 min, and re-equilibrated for 4 min. The flow rate was set to 0.5 mL min⁻¹, the injection volume 20 µL, and the column temperature was maintained at 40 °C.

The ESI source parameters used in positive mode were optimized as follows: spray voltage 3.5 kV; the capillary temperature was 250 °C; S-lens RF level 60 V; sheath gas flow rate at 47 L min^− 1^; and aux gas flow rate at 11 L min^− 1^. High-resolution mass spectra were acquired in the Full MS/data dependent -MS^2^ (dd–MS^2^) mode. The mass range in the full MS scanning experiments was *m/z* 100–1200. The top 5 (TopN, 5, loop count 5) most abundant precursors were sequentially transferred for collision-induced fragmentation acquisition. The collision energy for target analytes was 20, 30, and 35 eV. Resolving power was 140,000 and 70,000 for full MS and dd–MS^2^ acquisitions, respectively.

### Metabolite’s annotation

Metabolite’s annotation was performed using a combination of spectral matching and in silico tools available through the Global Natural Products Social Molecular Networking (GNPS) platform. The spectra data obtained from the LC–MS/MS analysis were first converted to the mzML format using MSConvert (ProteoWizard) and then processed with MZmine (v2.53) for feature detection and alignment. Annotation was primarily based on spectral matching against the GNPS MS/MS spectral libraries, which categorize matches according to spectral quality into gold (well-characterized standards), silver (tentative identification from crude extracts), and bronze (partial annotations) (Wang et al., 2016). Multiple GNPS tools were applied to enhance annotation coverage and confidence, including Classical Molecular Networking (MN) and Feature-Based Molecular Networking (FBMN) for visualization of structurally related metabolite clusters (Nothias et al., 2020; Wang et al., 2016), DEREPLICATOR + for in silico identification of peptide natural products (Mohimani et al., 2018), and Network Annotation Propagation (NAP) for structure propagation within molecular networks (da Silva et al. 2018). Additional tools such as MOLDISCOVERY (Cao et al., 2021), MS2LDA for substructure discovery (van der Hooft et al., 2016), and MolNetEnhancer for chemical class annotation were also employed (Ernst et al., 2019).

### Chemometric data analysis

The spectral data were extracted directly from the resulting.RAW files using the RawFileReader library (github.com/thermofisherlsms/RawFileReader), implemented in a custom Python3 script developed in-house. Following raw data extraction, the spectra were processed by discarding signal intensities below 1% of the maximum intensity within each scan, followed by spectral summation and base peak normalization, resulting in relative intensities from 0 to 100%. A mass alignment was performed across the samples, resulting in a final data matrix containing 113 rows (samples) and 1078 columns, each representing an *m/z* variable with its respective relative intensity. All statistical processing and data normalization were carried out using custom Python routines. This type of untargeted mass alignment has been shown to be effective for metabolomics analysis in complex plant systems (Gowda & Djukovic, 2014; Smith et al., 2006).

## Results and discussion

An untargeted metabolomics strategy was employed to investigate the molecular diversity of three Brazilian *Vanilla* species (*V. pompona*, *V. phaeantha*, and *V. calyculata* Schltr.) by analyzing ion abundance and metabolic composition using LC-HRMS/MS. To support metabolite annotation, dereplication procedures were employed by matching candidate structures within comprehensive spectral libraries, particularly those available through the GNPS platform. These analyses were complemented by in silico tools for MS/MS fragmentation prediction and structural elucidation, enhancing the reliability and depth of metabolite identification.

Leaf samples representing the four main Brazilian biomes (Cerrado, Amazon, Atlantic Forest, and Caatinga) were collected from individuals of the *Vanilla* Germplasm Bank at Embrapa Genetic Resources and Biotechnology, in Brasília, Federal District, for sample preparation. All information regarding species, biomes, and the number of samples are presented in the supporting information, Table S1.

The LC-HRMS/MS workflow included regular QC injections across all batches to assess reproducibility and monitor signal drift. All samples were spiked with two internal standards, and their retention times and ion intensities were monitored throughout the analysis. The consistency of these parameters confirmed the robustness and stability of the analytical platform throughout the measurements.

An initial investigation of the LC–MS dataset, which comprises 102 unique samples from three species (*V. pompona*, *V. phaeantha*, *V. calyculata* Schltr.) and four biomes, was performed using Principal Component Analysis (PCA). This unsupervised technique is designed to capture the maximum variance in the data, providing an unbiased view of its inherent structure (Bro & Smilde, 2014).

**Fig. 1:**
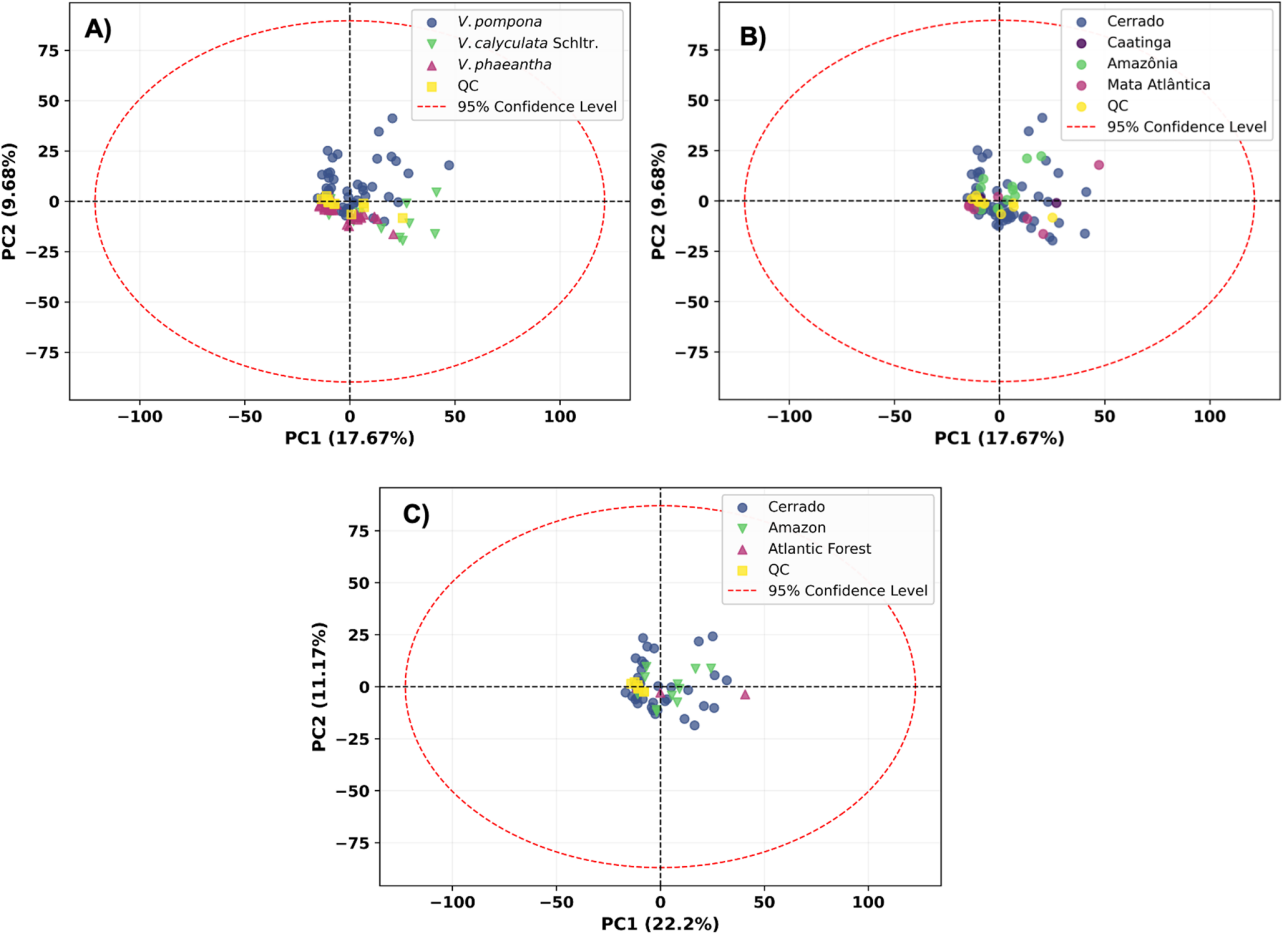
PCA scores plot of *Vanilla* samples. Samples are colored by **A** species, **B** biomes for all species, and **C** biomes for only *V. pompona*, showing a lack of natural clustering

The resulting PCA scores plot, shown in Fig. 1, reveals no distinct clustering of samples, either when colored by species (Fig. 1A), biomes for all species (Fig. 1B), or biomes for only *V. pompona* (the most abundant distribution) (Fig. 1C). The samples from different groups show extensive overlap, and the QC samples are tightly clustered near the origin, confirming the analytical stability of the LC–MS runs. The absence of spontaneous clustering among samples in the PCA highlights the predominance of intraspecific chemical variability in *Vanilla* leaves. This pattern is consistent with previous metabolomic investigations of *V. planifolia* leaves, which demonstrated that leaf chemistry is strongly influenced by ontogeny, photoperiod, and seasonal fluctuations, resulting in substantial intraindividual and intraspecific variation that can obscure taxonomic patterns (Palama et al., 2009). Such physiological plasticity inherent to leaf metabolism likely contributed to the broad chemical dispersion observed in our dataset, explaining why unsupervised multivariate methods were insufficient to resolve species-level groupings. Additionally, it is also important to note that the lack of clear species- or biome-specific grouping may be influenced by the fact that only leaf tissue was analyzed in this study. In *Vanilla* species, the characteristic aroma and many of the key secondary metabolites (e.g., vanillin and related phenolics) are predominantly synthesized and accumulated in the pods rather than in leaves, and metabolomic profiling of pods and mature beans has shown clear and statistically significant differences in metabolic composition compared to vegetative tissues (Beer et al., 2025; Gu et al., 2017). Previous studies have shown that developing and curing pods undergo pronounced biochemical transitions, accumulating glucovanillin, vanillin, and other phenylpropanoid derivatives far more intensely than leaves (Palama et al., 2009). Moreover, integrative metabolomic and transcriptomic work has demonstrated that key steps in vanillin biosynthesis, including those derived from ferulic acid, are strongly upregulated during pod maturation (Hernández-Peña et al., 2025). Thus, future analyses incorporating both pod and mature beans are likely to reveal more diagnostically powerful markers for both chemotaxonomy and flavor-related metabolic pathways that remain undetectable when only leaves are examined.

To deepen the analysis and focus on inter-species discrimination, a supervised approach using Partial Least Squares Discriminant Analysis (PLS-DA) was employed. Unlike PCA, PLS-DA is specifically designed to maximize the covariance between the spectral data (X-matrix) and a predefined class membership variable (Y-matrix), making it ideal for finding latent structures related to class differences (Barker & Rayens, 2003; Brereton & Lloyd, 2014; Lê Cao et al., 2011; Wold et al., 2001). Given the inherently binary nature of the PLS-DA algorithm, a *one-vs-all* strategy was employed to build separate models for species and biome classification. This approach mitigates the method’s multiclass limitations by decomposing the problem into multiple distinct two-class models (Brereton & Lloyd, 2014). Model optimization revealed that performance for all models was optimal using five latent components, as metrics plateaued thereafter (Figs. S1 and S2). The models designed to separate each species from the “Others” group yielded excellent results. Specifically, the *V. pompona* model showed outstanding predictive power (Q²=0.90), followed closely by the *V. phaeantha* model (Q²=0.82). The model for *V. calyculata* Schltr. was also robust and predictive (Q²=0.74). More details are shown in Fig. S1 and Table S2. These high Q² values, confirmed through leave-one-out cross-validation (LOOCV), indicate that the models identified true, generalizable chemical patterns for each species and were not merely overfitting the data (Neto et al., 2021; Triba et al., 2015). In the context of PLS-DA, Q² is a key metric for evaluating predictive ability because it directly reflects the cross-validated prediction error of the model. To further complement the performance assessment, Receiver Operating Characteristic (ROC) curves were also calculated (Xia et al., 2013). The resulting Area Under the Curve (AUC) values confirmed the strong classification capability of the models. The ROC curves are provided in the Supporting Information (Fig. S3). Despite this variability, supervised PLS-DA models achieved strong predictive performance for species discrimination, indicating that species-level chemical fingerprints do exist. Similar findings were reported by Leyva et al., (2021), who demonstrated that leaf metabolomes of *V. planifolia*, *V. pompona*, *V. palmarum*, and *V. ribeiroi* could be reliably discriminated through multivariate analyses despite their high intraspecific variance (Leyva et al., 2021).

In contrast, all models built to classify samples by biome failed to produce predictive results. Despite some high accuracy and R² values, their Q² values were consistently poor, ranging from − 0.19 to a maximum of only 0.22 for all biomes across species and from − 0.17 to 0.16 in the biome-based models restricted to *V. pompona*. Low or negative Q² values indicate poor predictive ability and suggest that the models do not capture a robust relationship between the data matrix and the biome classes. This finding strongly suggests that, while a distinct chemical fingerprint exists for each species, there is no consistent metabolic signature capable of reliably differentiating the biomes represented in this dataset (details in Fig. S2 and Table S2). These findings are also aligned with previous work showing that environmental origin explains far less variance in *Vanilla* leaves than endogenous physiological factors, which dominate the metabolomic landscape (Palama et al., 2009).

**Fig. 2:**
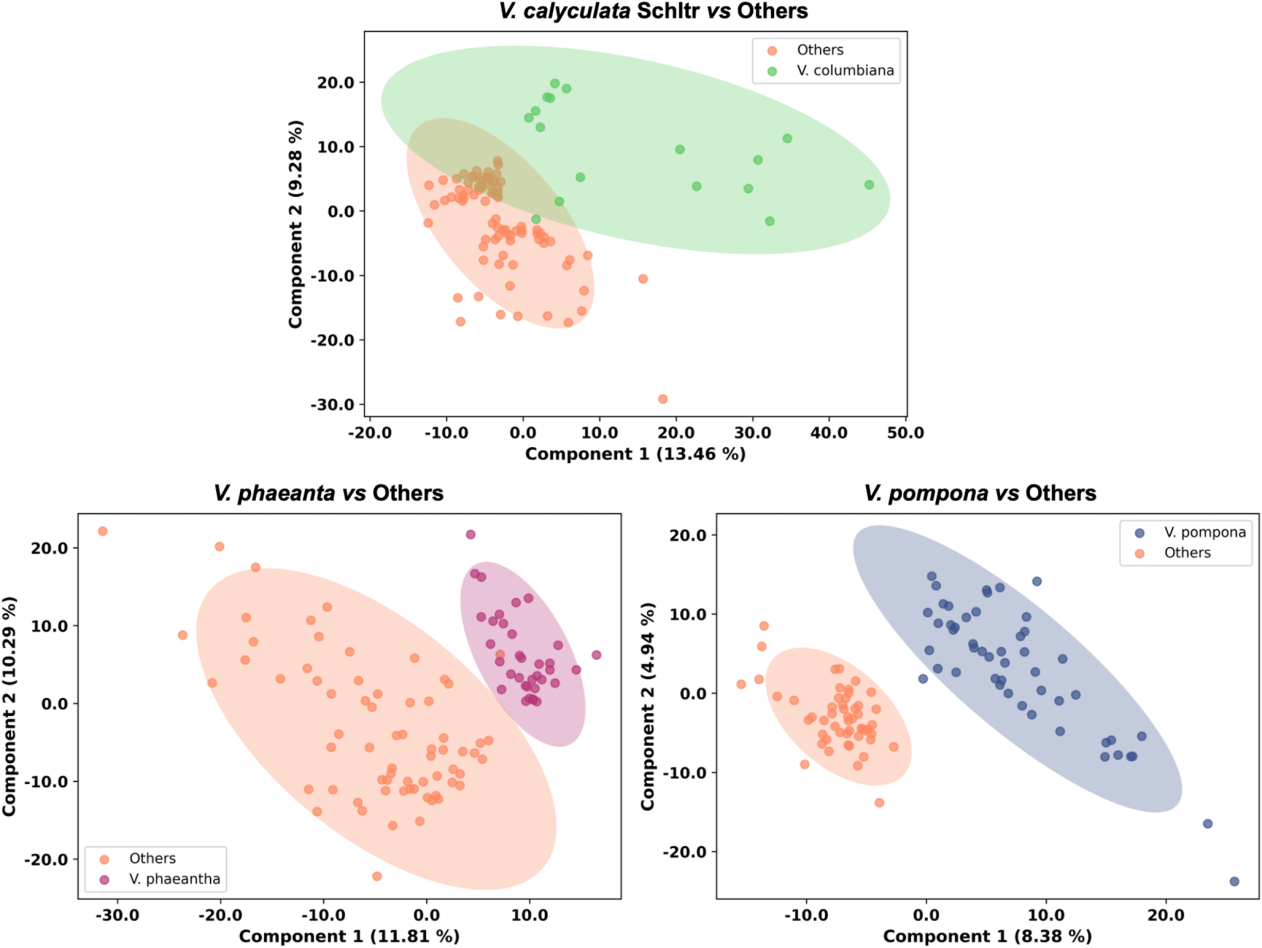
PLS-DA scores plot for species-based classification models, showing clear separations between the classes

The clear separation achieved by the species-specific models is visualized in the PLS-DA scores plots presented in Fig. 2. While all three models effectively discriminated the target species from the “Others” group, they did so with varying degree of distinction, a pattern consistent with their performance metrics. The model for *V. pompona* exhibited the most pronounced separation, corresponding to an accuracy of 100% using five components. Similarly, the *V. phaeantha* model showed a clear separation and achieved 99% accuracy. Although the model for *V. calyculata* Schltr. displayed the least distinct visual separation among the three, it remained highly effective, successfully differentiating the groups with 97% accuracy.

**Fig. 3:**
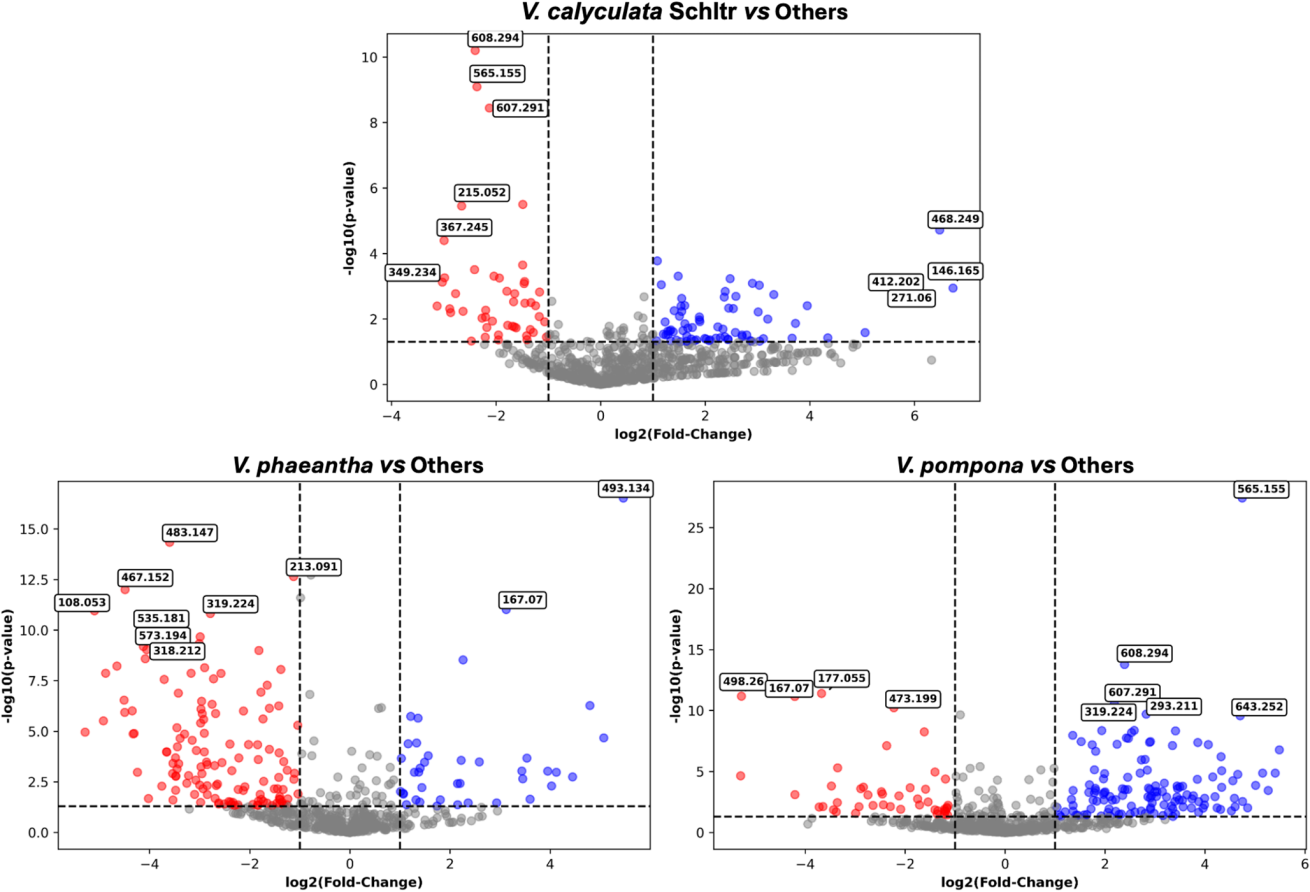
Volcano plots showing discriminant *m/z* features for each species comparison: *V. pompona vs.* “Others”, *V. phaeantha vs.*. “Others”, and *V. calyculata* Schltr. vs. “Others”. Each point represents an *m/z* feature. Features in the upper-right (more abundant in the target species) and upper-left (more abundant in the “Others”) quadrants are considered significantly different based on statistical significance (*p*-value) and magnitude of change (fold-change)

Having established that each species exhibits a robust chemical fingerprint, the next logical step was to identify the specific *m/z* features driving this discrimination. Discriminant variable selection was performed using a consensus-based strategy to ensure both biological relevance and statistical robustness. Although PLS-DA is well suited for addressing the multicollinearity characteristic of metabolomics datasets, Brereton and Lloyd (Brereton & Lloyd, 2014) caution that the method remains vulnerable to overfitting and the identification of spurious correlations in high-dimensional data. To mitigate these risks in feature selection, variable relevance was defined by the intersection of two complementary criteria: (i) multivariate importance, assessed through VIP scores > 1 in the PLS-DA models, as advocated by Wold et al. (Wold et al., 2001) for summarizing variable contribution in latent variable projections; and (ii) univariate significance and effect magnitude (fold-change), evaluated through Volcano Plot analysis, in accordance with established metabolomics protocols for identifying statistically significant features and distinguishing biological groups (Pan et al., 2021; Xu et al., 2024). This dual strategy ensures that selected metabolites contribute to the model’s global covariance structure (PLS) while also exhibiting statistically significant mean differences between groups, thereby aligning with recommendations that complex models be validated against the fundamental statistical properties of the dataset. Model robustness itself was assessed independently through leave-one-out cross-validation (LOOCV), used to calculate Q² values, and further supported by permutation testing to verify that the observed discrimination did not arise from random class assignments (Fig. S4). For each species-versus-others comparison, Volcano plots were constructed by plotting statistical significance (-log₁₀(*p*-value) against the magnitude of change (log₂(fold change). As shown in Fig. 3, this approach enables the rapid identification of features that are both statistically significant (high on the y-axis) and exhibit large relative differences between groups (far on the x-axis). To the further control for multiple hypothesis testing inherent to untargeted metabolomics datasets, the univariate p-values were subsequently corrected using the Benjamini-Hochberg false discovery rate (FDR) procedure (Benjamini & Hochberg, 1995). The complete table containing both raw p-values and FDR-adjusted q-values for all detected features is provided in the Supporting Information (Supplementary Spreadsheet), together with updated Volcano plots and Venn diagrams based on the q-value threshold (Fig. S5).

**Fig. 4:**
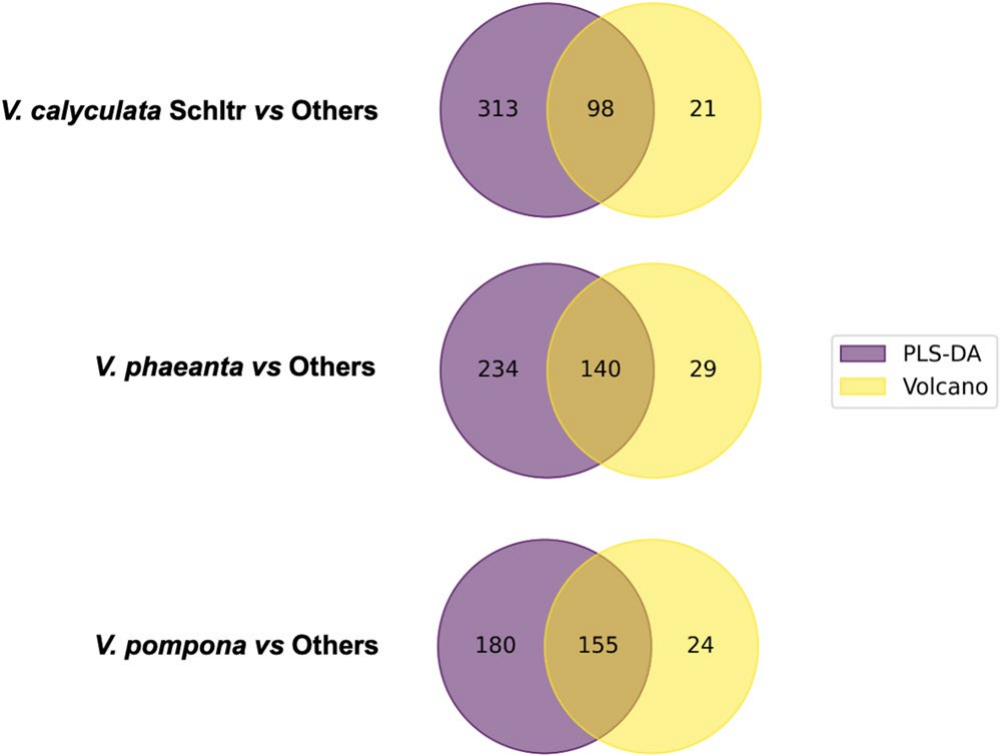
Venn diagrams show the intersection of discriminating *m/z* features for species-based datasets, identified by VIP score > 1.0 and Volcano plot significance criteria

To consolidate the findings from the univariate and multivariate analyses and identify the most reliable biomarkers, the list of significant features obtained from the Volcano Plots (*p* < 0.05 and |log₂(FC)| > 1) was then intersected with the list of variables deemed important by the PLS-DA model (VIP > 1.0). This combined filtering strategy ensures that selected metabolites not only contribute meaningfully to the model’s covariance structure but also display robust group-level differences. The overlap between these two feature sets was visualized using a Venn diagram (Fig. 4), highlighting the metabolites that consistently emerged as discriminant across both analytical frameworks. A similar combination of multivariate and univariate criteria has been shown to improve marker reliability in other metabolomic studies of *Vanilla*, supporting the robustness of the dual-filtering strategy employed here (Leyva et al., 2021).

The interpretation of this diagram is threefold.

(i) Intersection (VIP & Volcano): Features in this region represent the key biomarkers. They are validated as both statistically significant on their own and highly influential in the multivariate model, making them the highest-confidence candidates for differentiating the species.
(ii) VIP Only: Features in this region are important for the model’s predictive performance but lack strong individual statistical significance. Their contribution is driven by synergistic or combinatorial effects with other variables, underscoring the value of a multivariate perspective.
(iii) Volcano Only: Features in this region are statistically significant but were not prioritized by the PLS-DA model, possibly due to redundancy with other, more informative variables already captured by the model’s latent structure.

**Fig. 5:**
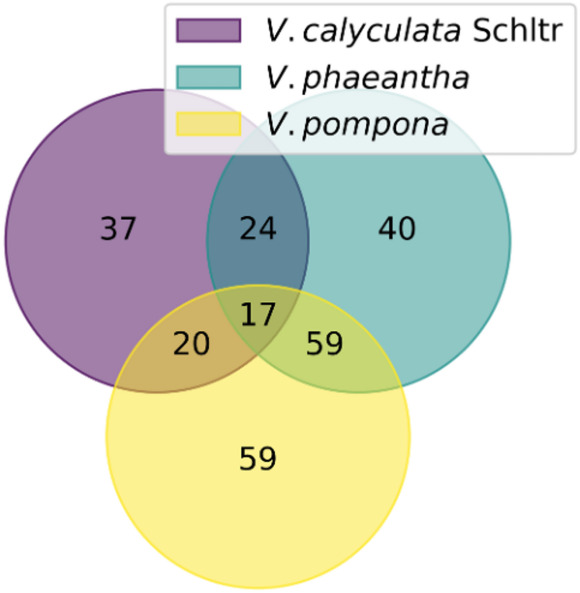
Three-way Venn diagram comparing the key biomarkers (features present in both VIP > 1.0 and Volcano Plot lists) for *V. calyculata* Schltr., *V. phaeantha*, and *V. Pompona*

Building on these results, we next examined the uniqueness and overlap of the chemical fingerprints across the three species. To do so, the key biomarkers (i.e., the intersection lists) identified for each species were compared using a three-way Venn diagram Fig. 5. This analysis reveals which markers are exclusive to a single species, serving as potential unique identifiers, and which are shared between two or all three species, potentially reflecting common biosynthetic pathways or structurally related metabolites. These patterns of shared and unique metabolites indicate that species differentiation in *Vanilla* arises from a combination of conserved metabolic scaffolds and species-specific modulations. Leyva et al., (2021) reported analogous trends, showing that Peruvian *Vanilla* species share core phenylpropanoid metabolites while differing in their relative abundances and in complementary pathways, supporting the idea of modular metabolic evolution within the genus (Leyva et al., 2021). This final step provides deeper insight into the chemical relationships among the species and yields a refined set of high-priority targets for structural elucidation.

The analysis of the three-way Venn diagram (Fig. 5) revealed a core set of 17 *m/z* features consistently identified as key molecular markers across all three species-specific models, indicating that these metabolites contribute fundamentally to the overall chemical differentiation among the studied *Vanilla* species leaves. Notably, ferulic acid (*m/z* 177.055 [M + H]^+^) and two esterified cinnamic acid derivatives—one conjugated to a terpenoid moiety (*m/z* 607.291 [M + H]^+^) and another linked to a nitrogen-containing chain (*m/z* 498.260 [M + Na]^+^)—emerged as central phenolic constituents. Additional shared discriminant features included the *C*-glycosylated flavonoid 6,8-di-*C*-glucopyranosylnaringenin (*m/z* 597.449 [M + H]^+^); several hydroxy fatty acid derivatives, represented by ions at *m/z* 277.216 [M + H]^+^ and 351.214 [M + Na]^+^; and a lipid species at *m/z* 318.248 [M + H]^+^, putatively assigned to a trihydroxylated sphingoid base. Further discriminant metabolites comprised an oxygenated polyunsaturated fatty acid derivative (*m/z* 293.211 [M + H]^+^), a lactone (*m/z* 367.245 [M + H]^+^), and a phenolic acid derivative (*m/z* 349.235 [M + H]^+^). In addition, three trihydroxylated kaurane-type diterpenoids (*m/z* 331.224, 333.240, and 335.219 [M + H]^+^), along with nitrogen-containing metabolites detected at *m/z* 336.222 and 608.294 [M + H]^+^ were identified as shared markers, together reflecting the interplay of multiple biosynthetic pathways in shaping species-specific chemical profiles.

**Fig. 6:**
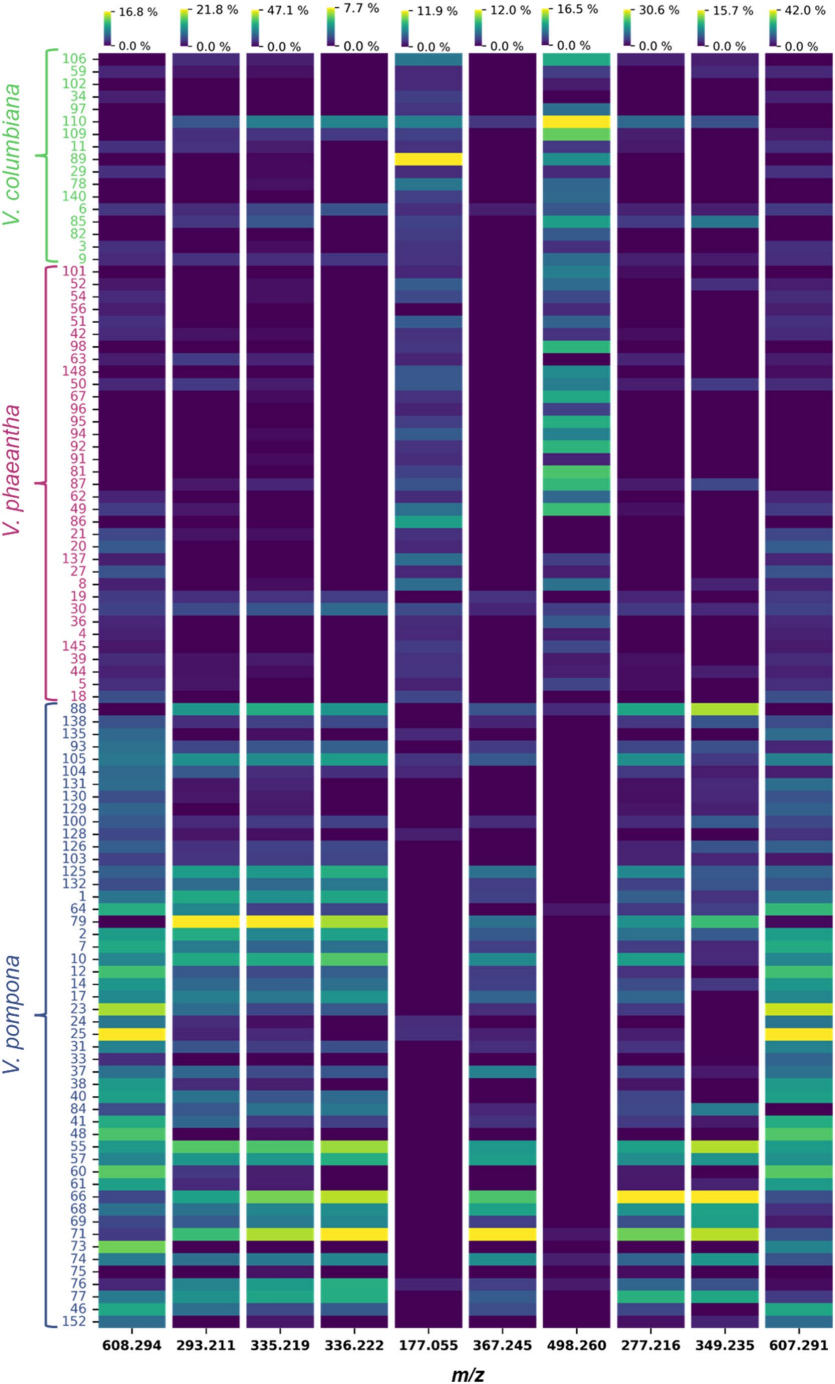
Heatmap of the top ten shared biomarkers with the highest VIP scores. Samples (rows) are grouped by species, and features (columns) are the selected *m/z* values. Colors represent normalized relative intensities, where each feature was independently scaled to highlight relative abundance patterns across species

Collectively, this metabolite set reinforces the central role of phenolic compounds, particularly phenolic acids and flavonoids, as fundamental secondary metabolites driving interspecific differentiation within the *Vanilla* genus. Among these shared features, ferulic acid emerged as a prominent marker. This finding has important biochemical implications, as ferulic acid is a confirmed precursor in the vanillin biosynthetic pathway, undergoing oxidative and reductive transformations that give rise to vanillin during pod development (Gallage & Møller, 2015; Kundu, 2017; Vogt, 2010). Its consistent detection across species suggests that vanillin-related phenylpropanoid metabolism constitutes a conserved biochemical axis within the genus. Similar observations were made by Leyva et al., (2021), who identified vanillin precursors (including glucosides A and B) as discriminant metabolites in the leaves of four *Vanilla* species, reinforcing the central chemotaxonomic role of phenolics. The prominence of flavonoids, terpenoids, lipid derivatives, and nitrogen-containing metabolites in our shared marker set further highlights the participation of multiple biosynthetic pathways in shaping interspecific differentiation. These compound classes have also been associated with adaptation, stress responses, and defense in *V. planifolia* and related taxa (Leyva et al., 2021; Palama et al., 2009). To further illustrate the abundance patterns of these shared discriminant ions, a heatmap was generated using the ten features with the highest VIP scores from this core set (Fig. 6). To further evaluate the clustering structure of the shared biomarkers, a hierarchical clustering heatmap using the full set of 17 discriminant ions is provided in the Supporting Information (Fig. S6). The complete list of detected features together with their corresponding statistical parameters (VIP scores, p-values, and fold changes) for each species vs. others comparison is provided in the Supporting Information as a supplementary spreadsheet.

**Fig. 7:**
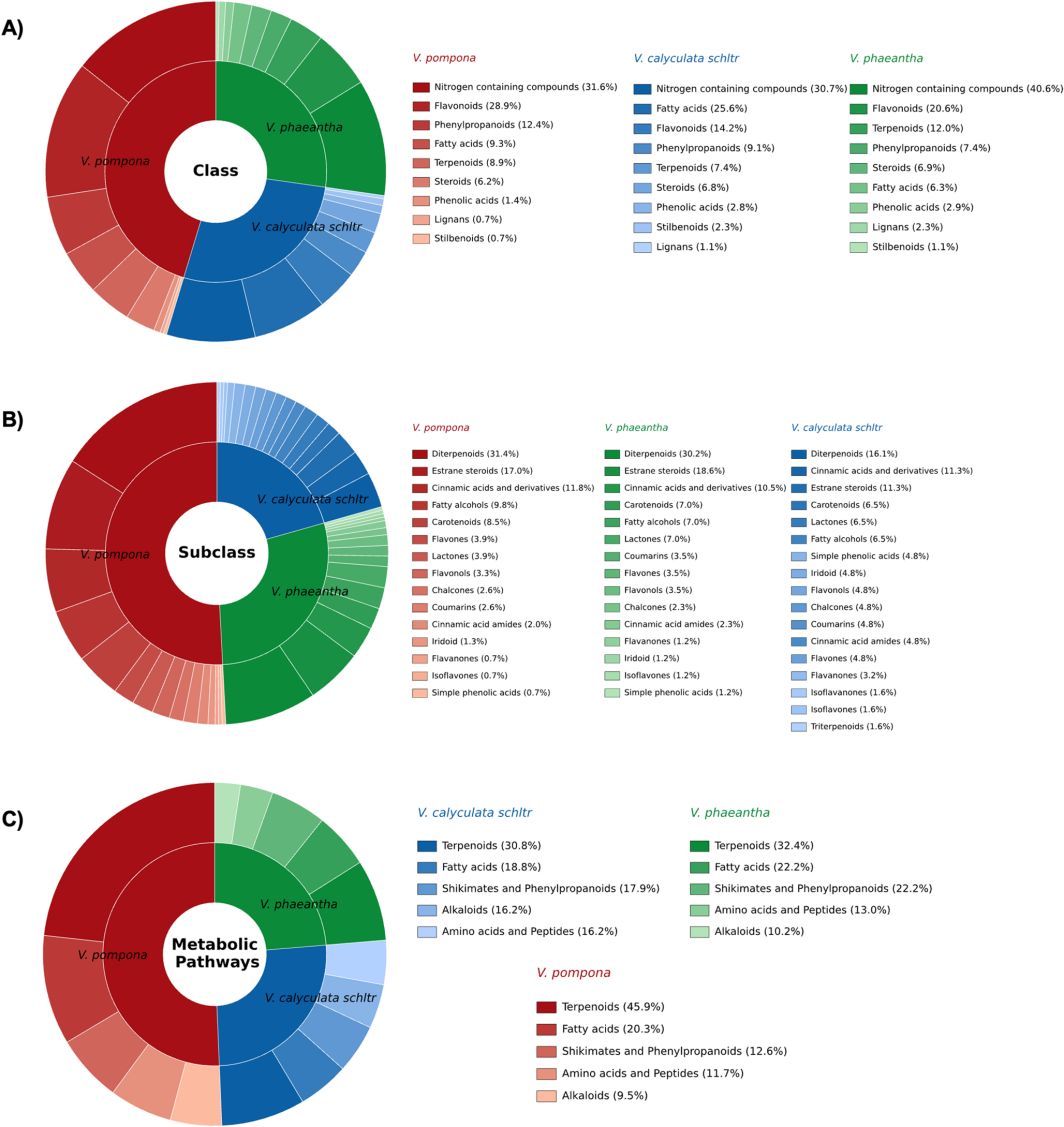
Classification of annotated metabolites in *V. calyculata* Schltr., *V. pompona*, and *V. phaeantha* based on MolNetEnhancer analysis. **A** Distribution across major chemical classes (flavonoids, terpenoids, lignans, steroids, phenylpropanoids, nitrogen-containing compounds, phenolic acids, fatty acids, stilbenoids), **B** subclass-level annotation (e.g., diterpenoids, sterane steroids, cinnamic acids and derivatives, flavones, flavonols, chalcones, carotenoids, coumarins, isoflavones), and **C** mapping to biosynthetic pathways (terpenoids, fatty acids, shikimates and phenylpropanoids, amino acids and peptides, alkaloids)

The class-level metabolite distribution highlighted clear chemical distinctions among the three *Vanilla* species (Fig. 7). Nitrogen-containing compounds emerged as the most abundant category across all taxa, particularly in *V. pompona*, suggesting a strong metabolic allocation to alkaloid- and peptide-related pathways in *Vanilla* genus (Dibwe et al., 2023). These compounds are often associated with ecological interactions such as defense and signaling, which may reflect adaptive strategies of *Vanilla* species to their natural habitats. Flavonoids represented another dominant class, with *V. pompona* (28.9%) again showing a markedly higher number of features than the other two species, followed by *V. phaeantha* (20.6%). This pattern underscores the importance of flavonoid metabolism for *Vanilla*, not only as pigments but also as antioxidants and modulators of plant–environment interactions (Beer et al., 2025). Metabolites classified as terpenoids displayed consistently low abundance across all samples (< 12%), a trend that may be attributed to their limited ionization efficiency under electrospray conditions, with detection being favored mainly for glycosylated derivatives. Within this context, *V. phaeantha* showed comparatively higher levels of this class. In contrast, phenylpropanoids, predominantly phenolic acids, were more abundant in *V. pompona* (12.4%), highlighting species-specific differences in the distribution of major secondary metabolite classes. Fatty acids, conversely, were particularly enriched in *V. calyculata* Schltr., setting this species apart from the others and indicating a distinct metabolic orientation towards primary-derived lipid pathways. Additional classes, such as terpenoids, steroids, phenylpropanoids, and lignans, were broadly shared but varied in their relative abundance, providing a baseline of conserved chemical traits with species-specific quantitative differences.

Subclass and pathway-level analyses further contextualized these observations (Fig. 7A and C). The enrichment of flavones, flavonols, and cinnamic acid derivatives in *V. pompona* points to a strong investment in the shikimate/phenylpropanoid pathway, consistent with its high number of annotated features in this metabolic route. *V. phaeantha* was distinguished by chalcones and carotenoids, reflecting specialized divisions of flavonoid and terpenoid biosynthesis that could reinforce its intermediate yet distinctive chemical profile. In contrast, *V. calyculata* Schltr. displayed a more balanced yet diverse chemical signature, with contributions from diterpenoids, flavones, and fatty acids, aligning with its dual enrichment in fatty acid and terpenoid pathways. Taken together, these results demonstrate that while the three *Vanilla* species share a common chemical composition, they diverge significantly in the intensity and diversification of specific metabolite classes. Such differences reinforce the discriminant statistical markers identified earlier and reveal the broader biosynthetic frameworks that shape inter-species metabolic variation within the genus.

## Conclusion

This study demonstrates the power of untargeted LC-HRMS/MS metabolomics combined with chemometric modeling to resolve the chemical diversity of Brazilian *Vanilla* species and to uncover robust species-specific metabolic fingerprints. Despite the pronounced intraspecific variability observed in leaf tissues, our integrative analytical strategy successfully discriminated *V. pompona*, *V. phaeantha*, and *V. calyculata* Schltr., revealing consistent multivariate patterns supported by high predictive power in supervised PLS-DA models. In contrast, the absence of biome-specific clustering underscores that environmental origin was not a major determinant of chemical variation under the conditions studied. By integrating multivariate (VIP scores) and univariate (Volcano Plot) criteria, we identified a high-confidence set of discriminant biomarkers, including phenolic acids, cinnamic acid derivatives, *C*-glycosylated flavonoids, lipids, terpenoids, and nitrogen-containing metabolites. The shared core of 17 markers, particularly ferulic acid (a biosynthetic precursor of vanillin) highlights central metabolic pathways conserved across these species and reinforces the relevance of phenylpropanoid metabolism in shaping *Vanilla* chemotaxonomy. Class-level and pathway-level annotations further revealed distinct metabolic orientations among the studied taxa, advancing our understanding of intra- and interspecific chemical differentiation within the genus. Together, these findings demonstrate that metabolomics offers a robust and a reliable tool for species authentication, chemotaxonomic studies, and the bioprospecting of aromatic and bioactive compounds in *Vanilla*. The molecular insights generated here provide a foundation for biodiversity conservation, germplasm management, and future valorization strategies involving Brazil’s native *Vanill*a species. Expanding future analyses to reproductive organs (green and cured pods) will reveal additional, diagnostically powerful metabolites directly linked to vanilla aroma biosynthesis and commercial quality.

## Supplementary Information

Below is the link to the electronic supplementary material.

Supplementary Material 1

Supplementary Material 2

## Data Availability

The mass spectrometric raw data supporting the findings will be made available by the authors upon reasonable request, without undue reservation.
