# Supplementary material for: Chemical diversity and species differentiation in Brazilian Vanilla: insights from LC-HRMS/MS metabolomics
Source: Metabolomics. 2026 Mar 28;22(2):48. doi: 10.1007/s11306-026-02422-8 (PMC13032978; doi:10.1007/s11306-026-02422-8)
Supplement: Supplementary file 1 — Supplementary Material 1 [file 11306_2026_2422_MOESM1_ESM.docx]

Supporting Information

Chemical Diversity and Species Differentiation in Brazilian Vanilla: Insights from LC-HRMS/MS Metabolomics

Gesiane S. Lima, ^1,2,#^ Giovanni B. Bevilaqua, ^1,#^ Hugo G. Machado, ^1,#^ Rosa B. N. Alves, ^3^ Luciano B. Bianchetti, ^3^ Lanaia I. L. Maciel, ^1^ Bianca M. M. G. Acioli, ^1^ Nerilson M. Lima, ^4^ Roberto F. Vieira, ^3,*^ Boniek Gontijo,^1^ Gabriel F. dos Santos ^1,*^

^1^ Institute of Chemistry, Federal University of Goiás, Goiânia, 59078-970, GO, Brazil

^2^ Department of Biological Sciences, State University of Santa Cruz, Ilhéus -BA, 45662-900, Brazil

^3^ Embrapa Recursos Genéticos e Biotecnologia, Parque Estação Biológica, Brasília, 70770-901, Distrito Federal, Brazil

^4^ Institute of Chemistry, Federal University of Alfenas, Alfenas, MG 37130-001, Brazil

#These authors contributed equally to this work

^*^ [roberto.vieira@embrapa.br](mailto:roberto.vieira@embrapa.br) (R. F. V.), [franco.gabriel@ufg.br](mailto:franco.gabriel@ufg.br) (G. F. S.)

**Table of contents:**

**Figure S1.** Bar charts of PLS-DA model performance metrics. The charts compare the accuracy, R², and Q² values for the three species-based models ………………………..**3**

**Figure S2.** Bar charts of PLS-DA model performance metrics. The charts compare the accuracy, R², and Q² values for A) four biome-based models, and B) three biome-based models for the specie *V. pompona*………………………………………………………..**4**

**Figure S3.** Receiver Operating Characteristics (ROC) curves for the PLS-DA species classification models. ROC analysis was performed for the three one-vs-others comparisons: (A) *V. calyculata* Schltr. vs. Others (AUC=0.95), (B) *V. phaeantha* vs. Others (AUC=0.99), and (C) *V. pompona* vs Others (AUC=1.00). The dashed line represents the performance expected for random classification. The high AUC values confirm the strong discriminative performance of the models in distinguishing each species based on their LC-HRMS metabolomic profiles……………………….……….**6**

**Figure S4.** Permutation tests for the PLS-DA classification models. Histograms represent the distribution of model performance obtained from permutations of class labels. The red dashed line indicates the observed model performance using the true class assignments. In all cases, the observed values lie outside the permutation distributions, supporting the statistical significance of the models and indicating that the discrimination is unlikely to arise from random structure in the data……………………………..……..**8**

**Figure S5.** Identification of discriminant ions after false discovery rate (FDR) correction. (A) Volcano plots for the comparisons (species vs. others). Each point represents an ion feature, with the x-axis showing log2 fold change (log2FC) and the y-axis showing -log10(q-value), where q-values were obtained using the Benjamini-Hochberg FDR correction. Features meeting the criteria q < 0.05 and |log2FC| > 1 are highlighted. Selected ions are labeled by their *m/z* values. (B) Venn diagrams showing the overlap between variables selected by PLS-DA (VIP > 1) and volcano plot analysis for each species comparison. (C) Venn diagram summarizing the overlap of discriminant ions among the three *Vanilla* species. After FDR correction, seven ions were consistently detected across the three species. ….................................................................................**5**

**Figure S5.** Heatmap with hierarchical clustering analysis (HCA) of the 17 shared discriminant ions identified across the species classification models. Rows represent individual samples and columns correspond to the selected m/z features. Hierarchical clustering was performed for both samples and variables, revealing grouping patterns consistent with species identity (*V. pompona*, *V. phaeantha*, and *V. calyculata* Schlt.). Colors represent normalized relative intensities, with higher abundances indicated by warmer colors………………………………………………………………..…………..**7**

**Table S1.** Biome origin for all *Vanilla* species (*V. pompona*, *V. phaeantha*, and *V. calyculata*) collected and used in this study….….............................................................**9**

**Table S2.** PLS-DA models performance metrics. Accuracy, R², and Q² values for the species-based models, biome-based models, and biome-based models restricted to *V. pompona*…………………………………………………………………….………...**10**

**Figures**

**
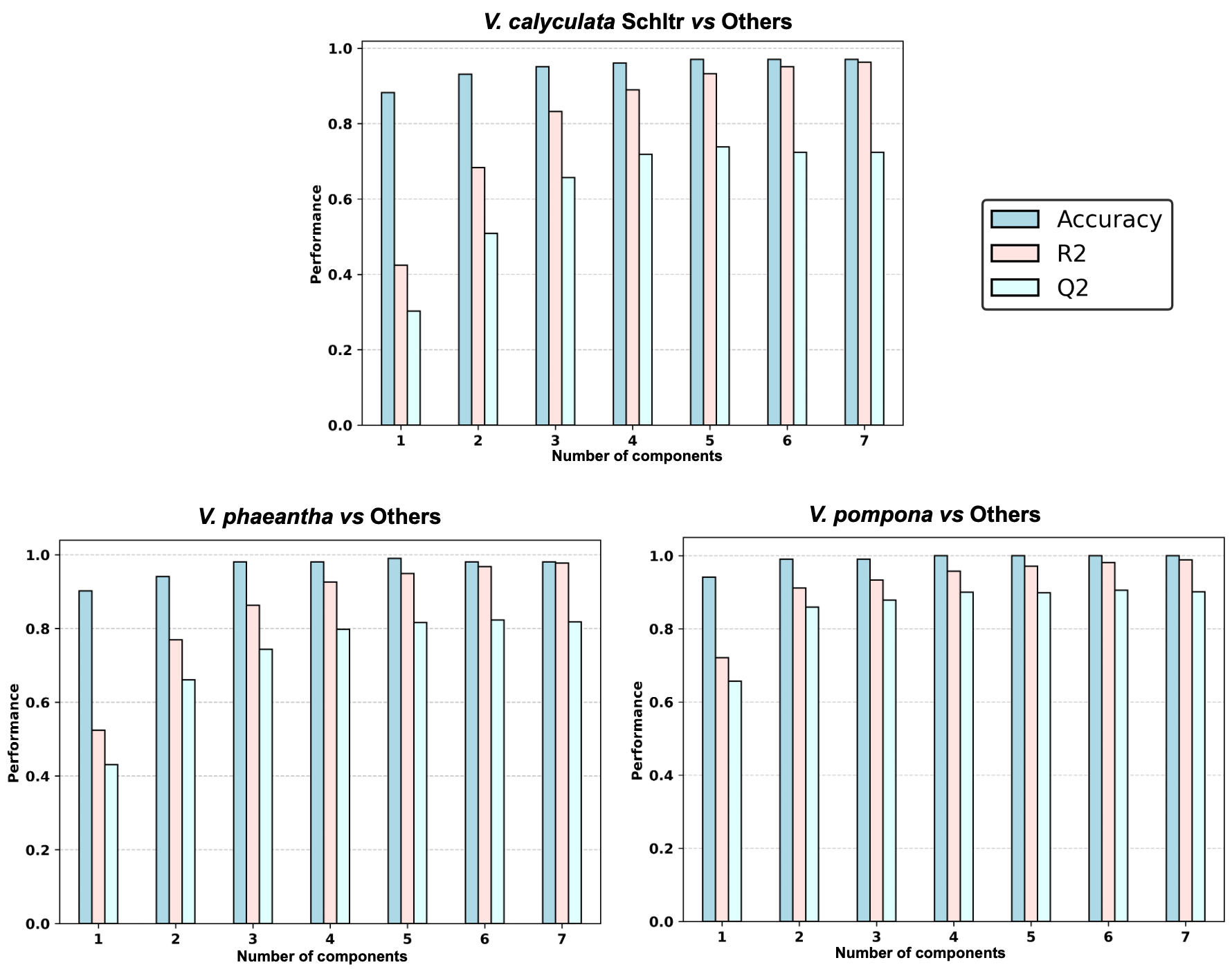
**

Figure S1. Bar charts of PLS-DA model performance metrics. The charts compare the accuracy, R², and Q² values for the three species-based models.


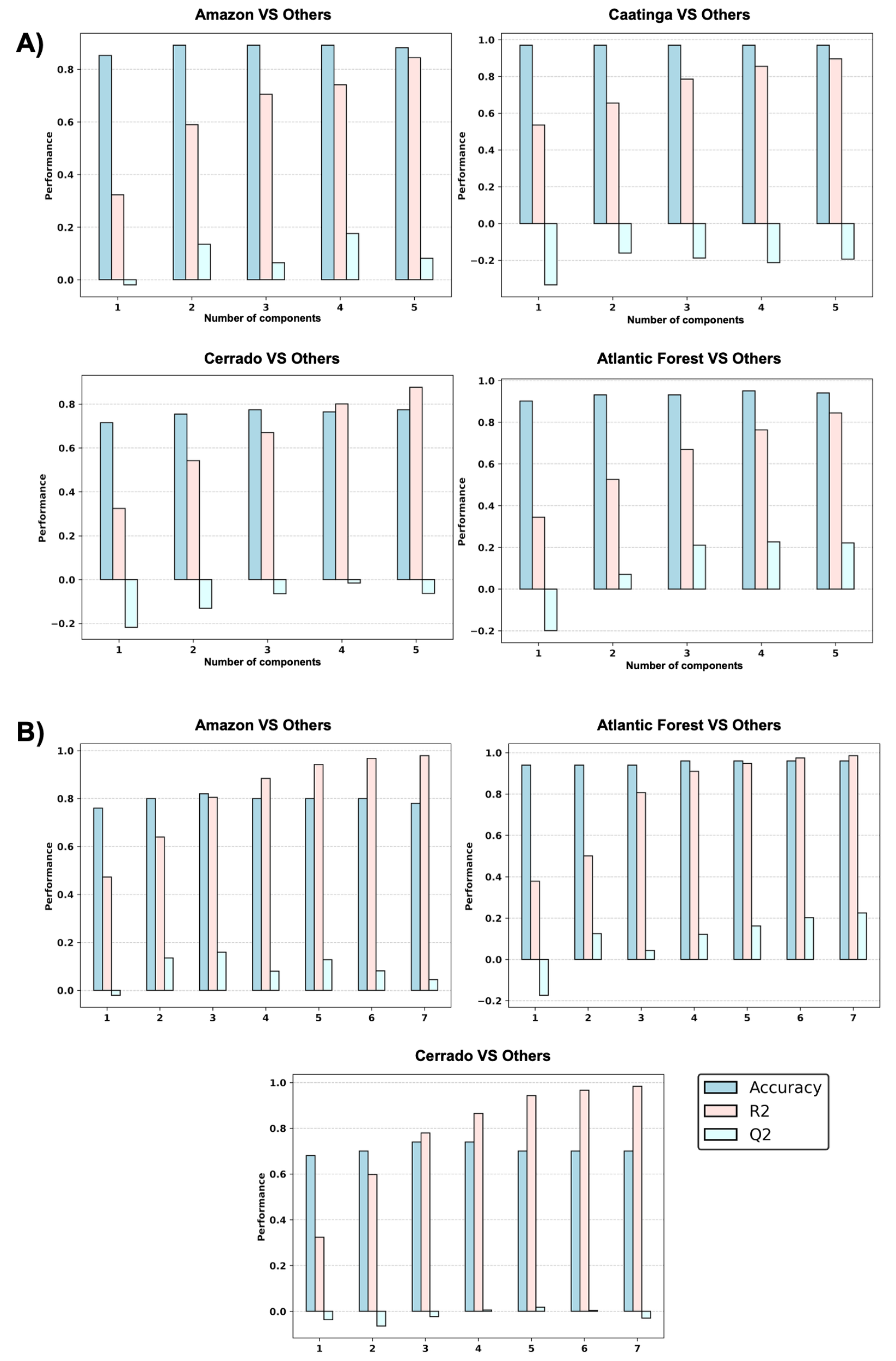


Figure S2. Bar charts of PLS-DA model performance metrics. The charts compare the accuracy, R², and Q² values for A) four biome-based models, and B) three biome-based models for the specie *V. pompona*.


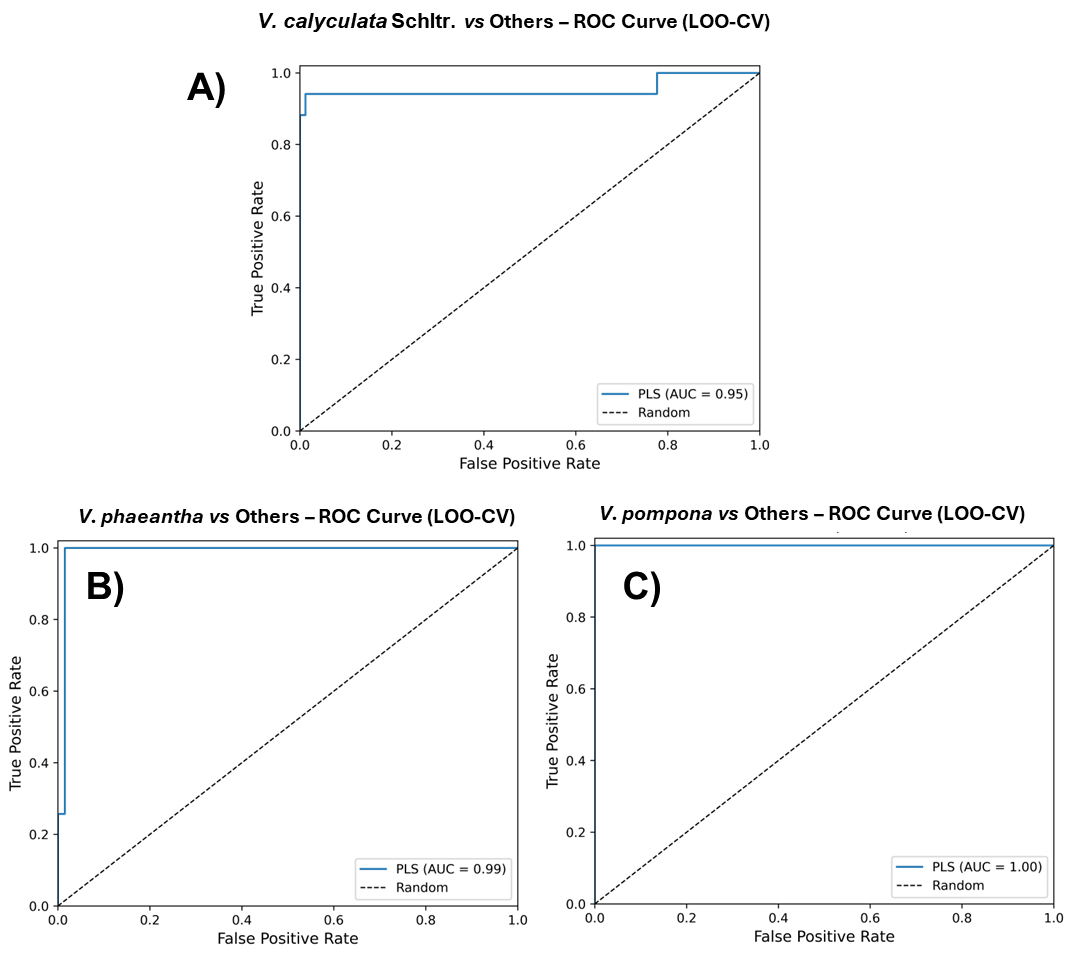


Figure S3. Receiver Operating Characteristics (ROC) curves for the PLS-DA species classification models. ROC analysis was performed for the three one-vs-others comparisons: (A) *V. calyculata* Schltr. *vs.* Others (AUC=0.95), (B) *V. phaeantha* *vs*. Others (AUC=0.99), and (C) *V. pompona* *vs* Others (AUC=1.00). The dashed line represents the performance expected for random classification. The high AUC values confirm the strong discriminative performance of the models in distinguishing each species based on their LC-HRMS metabolomic profiles.


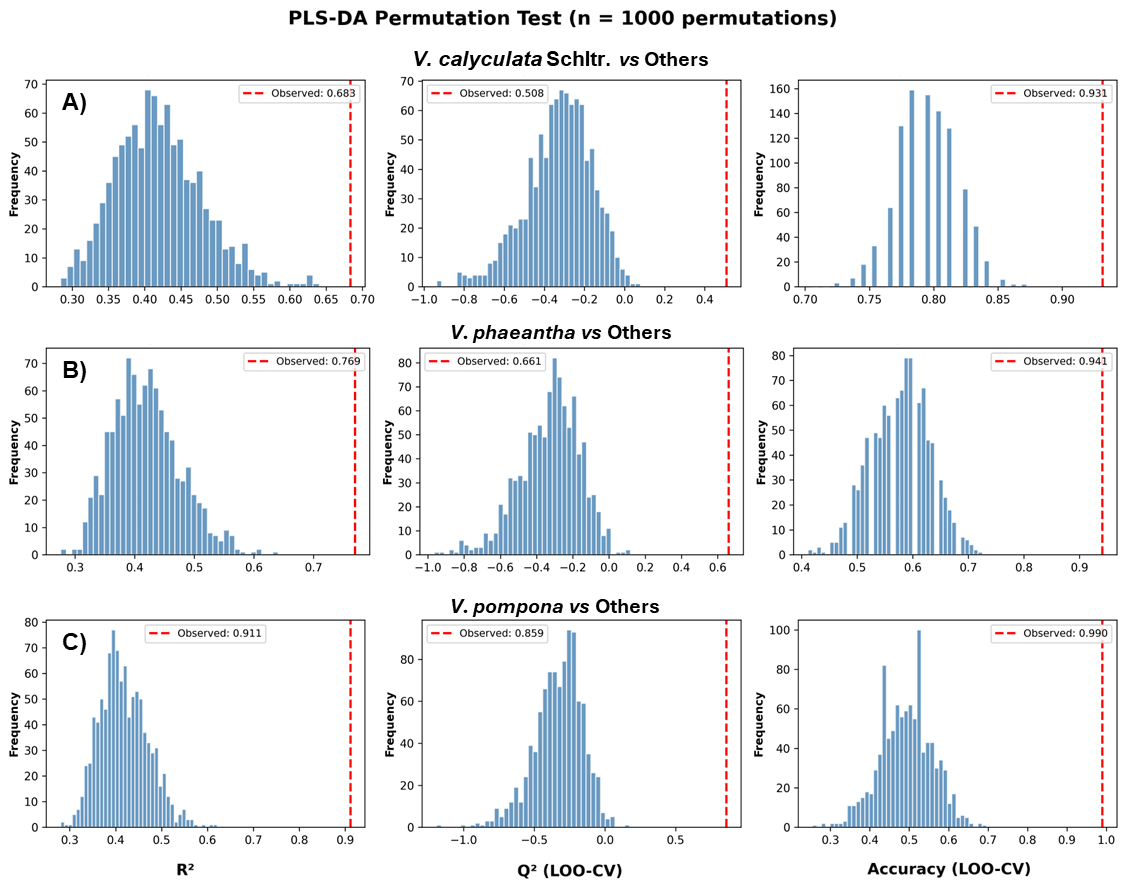


Figure S4. Permutation tests for the PLS-DA classification models. Histograms represent the distribution of model performance obtained from permutations of class labels. The red dashed line indicates the observed model performance using the true class assignments. In all cases, the observed values lie outside the permutation distributions, supporting the statistical significance of the models and indicating that the discrimination is unlikely to arise from random structure in the data.


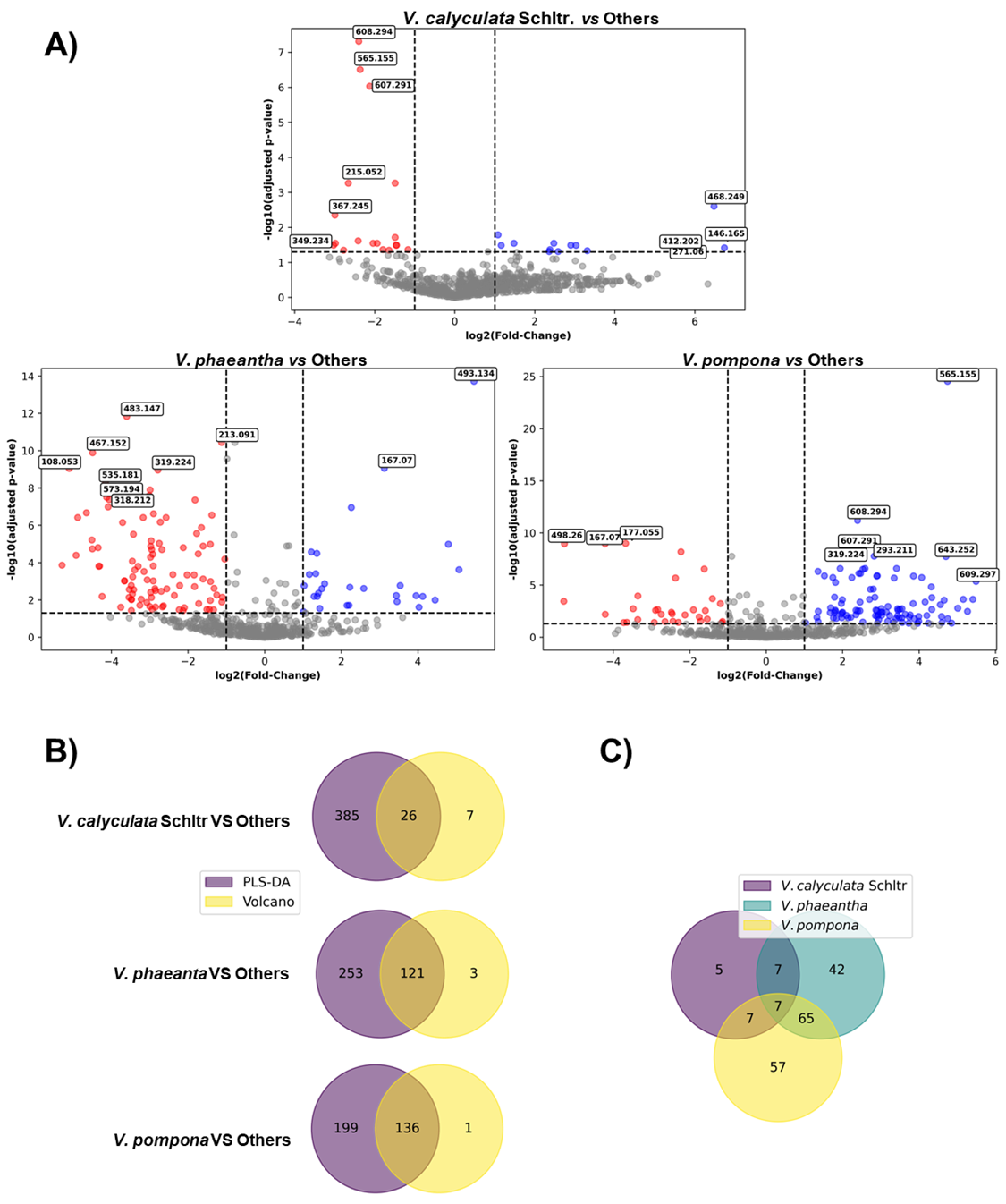


Figure S5. Identification of discriminant ions after false discovery rate (FDR) correction. (A) Volcano plots for the comparisons (*species vs. others*). Each point represents an ion feature, with the x-axis showing log_2_ fold change (log_2_FC) and the y-axis showing -log_10_(q-value), where q-values were obtained using the Benjamini-Hochberg FDR correction. Features meeting the criteria q < 0.05 and |log_2_FC| > 1 are highlighted. Selected ions are labeled by their *m/z* values. (B) Venn diagrams showing the overlap between variables selected by PLS-DA (VIP > 1) and volcano plot analysis for each species comparison. (C) Venn diagram summarizing the overlap of discriminant ions among the three Vanilla species. After FDR correction, seven ions were consistently detected across the three species.


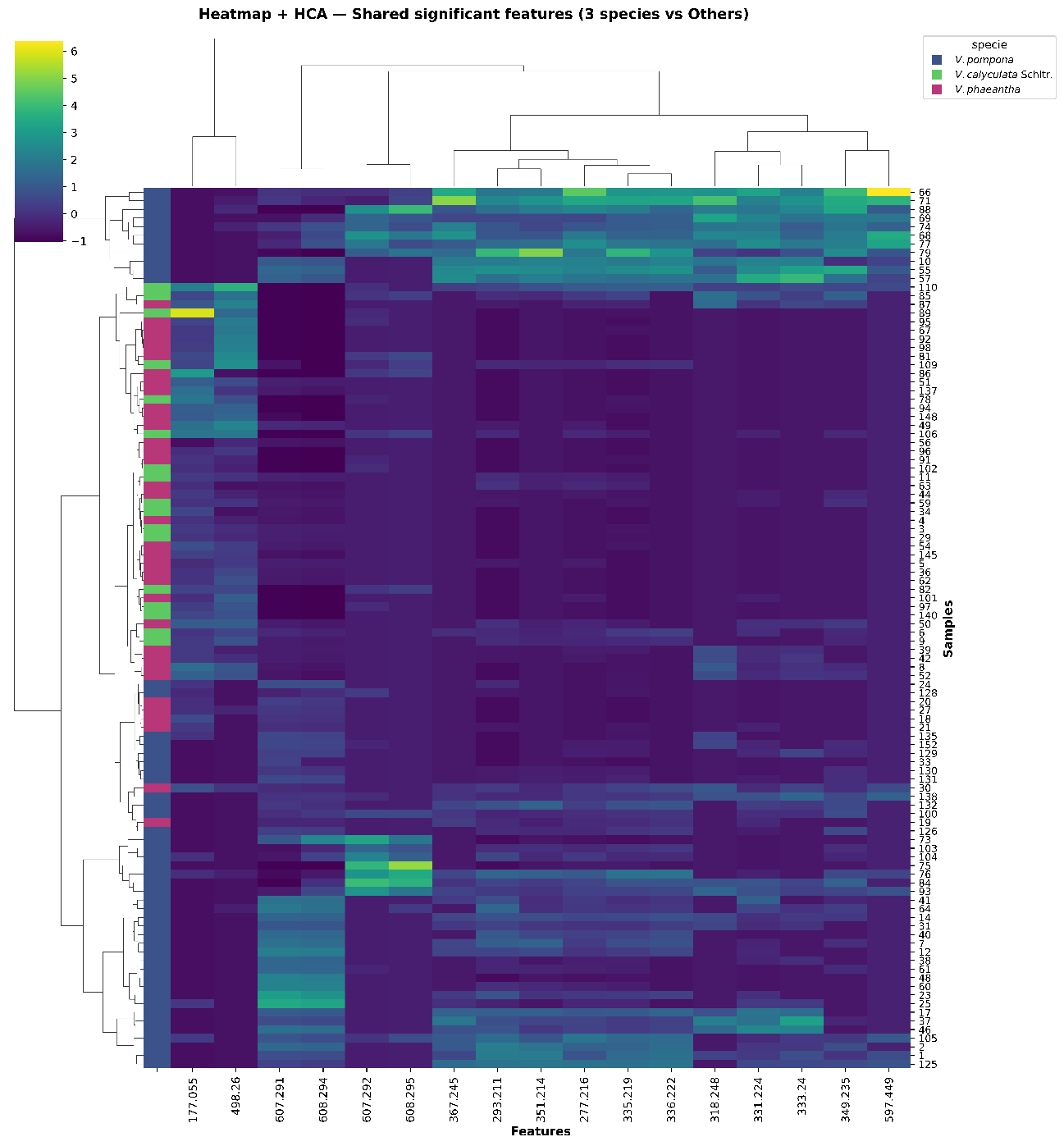


Figure S6. Heatmap with hierarchical clustering analysis (HCA) of the 17 shared discriminant ions identified across the species classification models. Rows represent individual samples and columns correspond to the selected *m/z* features. Hierarchical clustering was performed for both samples and variables, revealing grouping patterns consistent with species identity (*V. pompona*, *V. phaeantha*, and *V. calyculata* Schlt.). Colors represent normalized relative intensities, with higher abundances indicated by warmer colors.

**Tables**

Table S1. Biome origin for all *Vanilla* species (*V. pompona*, *V. phaeantha*, and *V. calyculata*) collected and used in this study

| **Code** | **Species** | **Biome** | **Code** | **Species** | **Biome** |
| --- | --- | --- | --- | --- | --- |
| A01 | *V. pompona* | Cerrado | A72 | *V. pompona* | Cerrado |
| A02 | *V. pompona* | Cerrado | A73 | *V. pompona* | Cerrado |
| A03 | *V calyculata* | Cerrado | A74 | *V. pompona* | Cerrado |
| A04 | *V. phaeantha* | Cerrado | A75 | *V. pompona* | Cerrado |
| A06 | *V calyculata* | Caatinga | A76 | *V. pompona* | Cerrado |
| A07 | *V. pompona* | Amazon | A77 | *V. pompona* | Amazon |
| A08 | *V. phaeantha* | Cerrado | A78 | *V calyculata* | Cerrado |
| A09 | *V calyculata* | Cerrado | A79 | *V. pompona* | Cerrado |
| A11 | *V calyculata* | Cerrado | A81 | *V. phaeantha* | Atlantic Forest |
| A12 | *V. pompona* | Cerrado | A82 | *V calyculata* | Cerrado |
| A14 | *V. pompona* | Cerrado | A83 | *V calyculata* | Cerrado |
| A17 | *V. pompona* | Cerrado | A84 | *V. pompona* | Cerrado |
| A18 | *V. phaeantha* | Atlantic Forest | A85 | *V calyculata* | Caatinga |
| A19 | *V. phaeantha* | Cerrado | A86 | *V. phaeantha* | Atlantic Forest |
| A20 | *V. phaeantha* | Atlantic Forest | A87 | *V. phaeantha* | Cerrado |
| A23 | *V. pompona* | Cerrado | A89 | *V calyculata* | Cerrado |
| A24 | *V. pompona* | Atlantic Forest | A91 | *V. phaeantha* | Cerrado |
| A25 | *V. pompona* | Cerrado | A93 | *V. pompona* | Amazon |
| A27 | *V. phaeantha* | Atlantic Forest | A94 | *V. phaeantha* | Cerrado |
| A28 | *V calyculata* | Cerrado | A95 | *V. phaeantha* | Cerrado |
| A29 | *V calyculata* | Caatinga | A96 | *V. phaeantha* | Cerrado |
| A30 | *V. phaeantha* | Cerrado | A97 | *V calyculata* | Cerrado |
| A33 | *V. pompona* | Amazon | A98 | *V. phaeantha* | Cerrado |
| A34 | *V calyculata* | Cerrado | A100 | *V. pompona* | Cerrado |
| A36 | *V. phaeantha* | Cerrado | A101 | *V. phaeantha* | Cerrado |
| A39 | *V. phaeantha* | Cerrado | A102 | *V calyculata* | Cerrado |
| A40 | *V. pompona* | Cerrado | A103 | *V. pompona* | Amazon |
| A40 | *V. pompona* | Cerrado | A104 | *V. pompona* | Amazon |
| A41 | *V. pompona* | Cerrado | A105 | *V. pompona* | Atlantic Forest |
| A42 | *V. phaeantha* | Amazon | A106 | *V calyculata* | Cerrado |
| A44 | *V. cf. phaeantha* | Cerrado | A107 | *V. pompona* | Cerrado |
| A49 | *V. phaeantha* | Atlantic Forest | A108 | *V. pompona* | Cerrado |
| A50 | *V. phaeantha* | Cerrado | A109 | *V calyculata* | Cerrado |
| A51 | *V. phaeantha* | Cerrado | A110 | *V calyculata* | Cerrado |
| A54 | *V. phaeantha* | Cerrado | A125 | *V. pompona* | Cerrado |
| A55 | *V. pompona* | Cerrado | A126 | *V. pompona* | Cerrado |
| A56 | *V. phaeantha* | Cerrado | A128 | *V. pompona* | Cerrado |
| A59 | *V. cf. calyculata* | Cerrado | A129 | *V. pompona* | Cerrado |
| A60 | *V. pompona* | Cerrado | A132 | *V. pompona* | Atlantic Forest |
| A62 | *V. phaeantha* | Cerrado | A135 | *V. pompona* | Amazon |
| A63 | *V. phaeantha* | Cerrado | A137 | *V. phaeantha* | Cerrado |
| A64 | *V. pompona* | Cerrado | A138 | *V. pompona* | Cerrado |
| A66 | *V. pompona* | Cerrado | A140 | *V. cf. calyculata* | Cerrado |
| A67 | *V. phaeantha* | Cerrado | A145 | *V. phaeantha* | Cerrado |
| A68 | *V. pompona* | Amazon | A148 | *V. phaeantha* | Cerrado |
| A69 | *V. pompona* | Amazon | A152 | *V. pompona* | Amazon |

Table S2. PLS-DA models performance metrics. Accuracy, R², and Q² values for the species-based models, biome-based models, and biome-based models restricted to *V. pompona*

| **PLS-DA Model** | **Number of components** | **Accuracy** | **R²** | **Q²** |
| --- | --- | --- | --- | --- |
| ***V. calyculata* *vs* Others** | 1 | 0.88 | 0.42 | 0.30 |
|  | 2 | 0.93 | 0.68 | 0.51 |
|  | 3 | 0.95 | 0.83 | 0.66 |
|  | 4 | 0.96 | 0.89 | 0.72 |
|  | 5 | 0.97 | 0.93 | 0.74 |
| ***V. phaeantha* *vs* Others** | 1 | 0.90 | 0.52 | 0.43 |
|  | 2 | 0.94 | 0.77 | 0.66 |
|  | 3 | 0.98 | 0.86 | 0.74 |
|  | 4 | 0.98 | 0.93 | 0.80 |
|  | 5 | 0.99 | 0.95 | 0.82 |
| ***V. pompona* *vs* Others** | 1 | 0.94 | 0.72 | 0.66 |
|  | 2 | 0.99 | 0.91 | 0.86 |
|  | 3 | 0.99 | 0.93 | 0.88 |
|  | 4 | 1.00 | 0.96 | 0.90 |
|  | 5 | 1.00 | 0.97 | 0.90 |
| **Cerrado *vs* Others**  **(all three species)** | 1 | 0.72 | 0.32 | -0.22 |
|  | 2 | 0.75 | 0.54 | -0.13 |
|  | 3 | 0.77 | 0.67 | -0.06 |
|  | 4 | 0.76 | 0.80 | -0.02 |
|  | 5 | 0.77 | 0.88 | -0.06 |
| **Amazon *vs* Others**  **(all three species)** | 1 | 0.85 | 0.32 | -0.02 |
|  | 2 | 0.89 | 0.59 | 0.14 |
|  | 3 | 0.89 | 0.71 | 0.06 |
|  | 4 | 0.89 | 0.74 | 0.18 |
|  | 5 | 0.88 | 0.84 | 0.08 |
| **Atlantic Forest *vs* Others**  **(all three species)** | 1 | 0.90 | 0.34 | -0.20 |
|  | 2 | 0.93 | 0.52 | 0.07 |
|  | 3 | 0.93 | 0.67 | 0.21 |
|  | 4 | 0.95 | 0.76 | 0.23 |
|  | 5 | 0.94 | 0.84 | 0.22 |
| **Caatinga *vs* Others**  **(all three species)** | 1 | 0.97 | 0.54 | -0.33 |
|  | 2 | 0.97 | 0.65 | -0.16 |
|  | 3 | 0.97 | 0.79 | -0.19 |
|  | 4 | 0.97 | 0.86 | -0.21 |
|  | 5 | 0.97 | 0.90 | -0.19 |
| **Amazon *vs* Others**  **(restricted to *V. pompona*)** | 1 | 0.76 | 0.47 | -0.02 |
|  | 2 | 0.80 | 0.64 | 0.14 |
|  | 3 | 0.82 | 0.80 | 0.16 |
|  | 4 | 0.80 | 0.88 | 0.08 |
|  | 5 | 0.80 | 0.94 | 0.13 |
| **Atlantic Forest *vs* Others**  **(restricted to *V. pompona*)** | 1 | 0.94 | 0.38 | -.017 |
|  | 2 | 0.94 | 0.50 | 0.12 |
|  | 3 | 0.94 | 0.81 | 0.04 |
|  | 4 | 0.96 | 0.91 | 0.12 |
|  | 5 | 0.96 | 0.95 | 0.16 |
| **Cerrado *vs* Others**  **(restricted to *V. pompona*)** | 1 | 0.68 | 0.32 | -0.04 |
|  | 2 | 0.70 | 0.60 | -0.06 |
|  | 3 | 0.74 | 0.78 | -0.02 |
|  | 4 | 0.74 | 0.86 | 0.01 |
|  | 5 | 0.70 | 0.94 | 0.00 |
